# Bronchiectasis, the Latest Eosinophilic Airway Disease: What about the Microbiome?

**DOI:** 10.1164/rccm.202201-0105ED

**Published:** 2022-02-25

**Authors:** Dave Singh, Chris Brightling

**Affiliations:** ^1^ University of Manchester Manchester University NHS Foundation Trust Manchester, United Kingdom; ^2^ Department of Respiratory Sciences University of Leicester Leicester, United Kingdom

Asthma and chronic obstructive pulmonary disease (COPD) are heterogeneous conditions in which biomarkers can help identify individuals who require different management strategies. In asthma, higher type 2 biomarker measurements, including blood eosinophil counts (BECs), can identify individuals with a greater likelihood of a positive corticosteroid response or those with severe asthma who are candidates for biological therapies directed against type 2 inflammation (1).

The Global Initiative for Chronic Obstructive Lung Disease management strategy supports the use of BECs in patients with COPD with increased exacerbation risk to help direct appropriate inhaled corticosteroid (ICS) use (2). The relationship between BEC and ICS effects is continuous; a BEC <100 cells/μl identifies individuals with no or little possibility of ICS benefit, with the likelihood of clinical benefit increasing incrementally above this threshold. Higher BECs are associated with increased levels of type 2 inflammation in COPD lungs (3), which can explain the differential ICS response between individuals. Recent studies have demonstrated that patients with COPD with lower BECs and sputum eosinophil counts during the stable state have greater abundance of proteobacteria, including *Haemophilus*, in the airways (4, 5) and that lower BECs are associated with increased risk of bacterial infections and pneumonia (6). When used alongside other clinical information, BECs now appear to be a COPD biomarker that can help identify subgroups with differential pharmacological treatment responses or a different microbiome.

Attention is now placed on BEC in bronchiectasis because a subset display sputum eosinophilia (⩾3%) (7). A clinical trial *post hoc* analysis suggested that greater improvements in quality of life with ICS occurred at higher BECs in patients with bronchiectasis (8). These findings suggest the biomarker potential of BEC in bronchiectasis.

In this issue of the *Journal*, Shoemark and colleagues (pp. 894–902) analyzed data from different bronchiectasis cohorts to further evaluate the biomarker potential of BECs (9). First, a significant but not strong association (*r* = 0.31; *P *< 0.0001) was demonstrated between BECs and sputum eosinophil counts (*n *= 235). This is similar to the weak to moderate strength correlations in asthma and COPD studies, reflecting between-day and within-day variability and measurement method variability (10). If we are content to use BECs as a surrogate of lung eosinophil numbers in asthma and COPD, then these results support the same for bronchiectasis.

Next, data from the FRIENDS (Facilitating Research Into Existing National Datasets) cohort (*n* = 951) was stratified using the BEC thresholds <100, 100–299, and ⩾300 cells/μl. There were no differences in exacerbation rates or mortality over 12 months, even after accounting for possible ICS effects. The authors then investigated the sputum microbiome by 16S rRNA sequencing (*n* = 198). BECs <100 cells/μl were associated with *Haemophilus*- and *Moraxella*-predominant microbiomes, whereas BECs ⩾300 cells/μl were associated with a *Streptococcus*-predominant microbiome. Intriguingly, *Pseudomonas* had a more complex relationship with associations in the eosinophil-high group, as stated by the authors, but also was apparent in the eosinophil-low group when defining individuals according to the dominant organism. The *Haemophilus*-dominant/low-eosinophil subgroup has been described in asthma and COPD (4, 11), suggesting that common relationships exist across different airway diseases between the microbiome and inflammation characteristics.

Finally, data from a clinical trial of inhaled colistin versus placebo for 6 months was used to evaluate the relationship between exacerbations and BECs in patients with bronchiectasis whose infection status had been homogenized and controlled for by including only individuals with *Pseudomonas aeruginosa* colonization who had recently received antipseudomonal antibiotics. The time to first exacerbation was shorter in patients with baseline BECs ⩾100 versus <100 cells/μl after adjusting for baseline confounding factors, including bacterial load, and was shortest in those with BECs ⩾300 cells/μl. This relationship between exacerbation risk and BEC was present in both treatment arms, and BECs were stable before and after treatment in each arm. These results implicate eosinophilic inflammation in the pathophysiology of some bronchiectasis exacerbations and indicate that BEC is a potential risk biomarker in this context, but they do not suggest that attenuating bacterial load modifies eosinophilic inflammation. Furthermore, these conclusions can be applied only to the subgroup of individuals with *P. aeruginosa* infection. Critically, a clinical trial showed that *P. aeruginosa* bacterial density was substantially reduced by colistin, but the effect on exacerbation frequency was less convincing than the marked benefit for quality of life (12). This suggests that targeting microbial dysbiosis or eosinophilic inflammation might differentially affect clinical outcomes and could be different for different microbiome predominance. The failure to find a relationship between BEC and exacerbation risk in the FRIENDS cohort is likely due, at least partly, to confounding by bacterial infection in a heterogeneous population. Insufficient control for confounding factors, including the microbiome, can similarly explain other discordant results from recent bronchiectasis cohorts; higher BECs (>100 cells/μl) have been associated with milder disease characteristics, whereas higher T2 biomarkers, either BEC ⩾300 cells/μl or higher exhaled nitric oxide levels, have been associated with more severe disease characteristics (13, 14). Reduced microbiome diversity has been associated with worse clinical outcomes in bronchiectasis, with a *Pseudomonas*-dominant microbiome associated with more exacerbations and worse mortality (15). Shoemark and colleagues demonstrated relationships between microbiome characteristics and BEC, reinforcing that BEC is not a stand-alone biomarker in bronchiectasis but one that can be interpreted only in combination with microbiome information.

The findings of Shoemark and colleagues confirm that eosinophilic inflammation is an important feature, albeit in a minority of patients with bronchiectasis, consistent with findings in COPD and in contrast to the archetypal eosinophilic airway disease asthma. This illustrates that eosinophilic inflammation plays a role across the spectrum of different airway diseases. Importantly, in those with bronchiectasis, eosinophilic inflammation was associated with higher exacerbation frequency after accounting for infection status as a confounder. Eosinophil-targeted therapy might be beneficial in a subgroup of people with bronchiectasis, but airway inflammation and microbial dysbiosis must be considered in concert rather than in isolation. There is a need to further explore the mechanisms driving the different microbiome composition observed in those with and without eosinophilic inflammation and the consequent impact on clinical management.
